# Ryanodine receptors: physiological function and deregulation in Alzheimer disease

**DOI:** 10.1186/1750-1326-9-21

**Published:** 2014-06-05

**Authors:** Dolores Del Prete, Frédéric Checler, Mounia Chami

**Affiliations:** 1 Université de Nice Sophia Antipolis, IPMC, Sophia Antipolis, Nice, F-06560 Valbonne, France; 2 CNRS, IPMC, Sophia Antipolis, Nice, F-06560 Valbonne, France; 3 Present address: Albert Einstein College of Medicine, Bronx, New York 10461NY, USA

**Keywords:** Ryanodine receptor, Calcium, Alzheimer disease, Endoplasmic reticulum, Neurodegeneration, Presenilin, Amyloid precursor protein, Amyloid beta

## Abstract

Perturbed Endoplasmic Reticulum (ER) calcium (Ca^2+^) homeostasis emerges as a central player in Alzheimer disease (AD). Accordingly, different studies have reported alterations of the expression and the function of Ryanodine Receptors (RyR) in human AD-affected brains, in cells expressing familial AD-linked mutations on the β amyloid precursor protein (βAPP) and presenilins (the catalytic core in γ-secretase complexes cleaving the βAPP, thereby generating amyloid β (Aβ) peptides), as well as in the brain of various transgenic AD mice models. Data converge to suggest that RyR expression and function alteration are associated to AD pathogenesis through the control of: i) βAPP processing and Aβ peptide production, ii) neuronal death; iii) synaptic function; and iv) memory and learning abilities. In this review, we document the network of evidences suggesting that RyR could play a complex dual “compensatory/protective *versus* pathogenic” role contributing to the setting of histopathological lesions and synaptic deficits that are associated with the disease stages. We also discuss the possible mechanisms underlying RyR expression and function alterations in AD. Finally, we review recent publications showing that drug-targeting blockade of RyR and genetic manipulation of RyR reduces Aβ production, stabilizes synaptic transmission, and prevents learning and memory deficits in various AD mouse models. Chemically-designed RyR “modulators” could therefore be envisioned as new therapeutic compounds able to delay or block the progression of AD.

## Introduction

**Figure 1:**
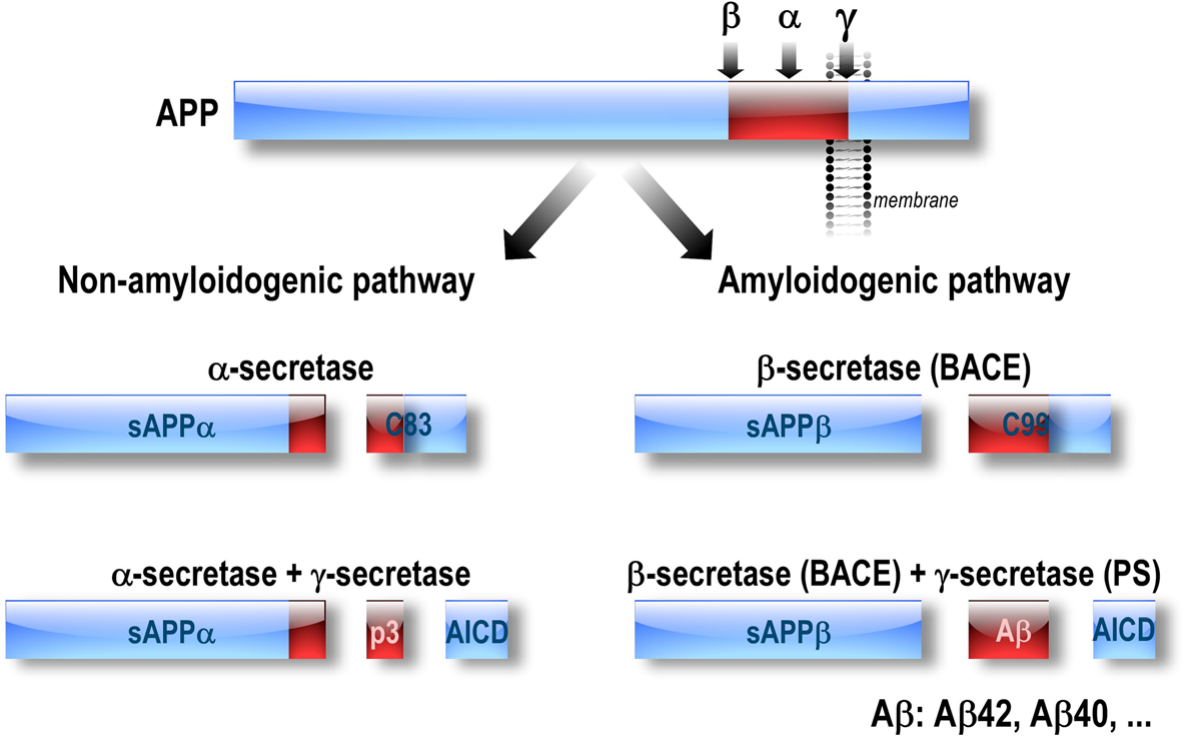
**Amyloidogenic and non-amyloidogenic pathways of β amyloid precursor protein (βAPP) processing.** Aβ peptides are derived from the processing of the βAPP through the amyloidogenic pathway, implicating β-secretase (BACE1) and γ-secretase complex (composed of PS1 or PS2, Nicastrin, anterior pharynx-defective-1 (APH-1), and presenilin enhancer-2 (PEN-2)). Aβ production is abolished when βAPP is processed through the non-amyloidogenic pathway implicating α-secretase and γ-secretase complexes.

Alzheimer Disease (AD) is the most common type of dementia characterized clinically by progressive deterioration of cognitive functions including memory, reasoning, and language [1]. Neuropathological hallmarks of the disease include extracellular amyloid plaques mainly composed of a set of hydrophobic peptides referred to as β-amyloid peptides (Aβ) aggregates and intracellular neurofibrillar tangles (NFT) composed of hyperphosphorylated microtubule-associated tau protein [2-4]. Aging is the major risk factor for the most common late-onset cases AD. However, a significant number of aggressive cases generally characterized by an earlier onset are inherited in an autosomal dominant manner (FAD) [5,6], and caused by mutations on the β-Amyloid precursor protein (βAPP, the precursor of the Aβ peptides) [7] and on presenilins (PS1 and PS2) (catalytic core components of the βAPP cleaving enzyme γ-secretase [2,3]) (Figure 1). Interestingly, both mutations in PS1-2 and βAPP proteins either modify the nature of Aβ peptides and/or affect the levels of their production [8,9].

**Figure 2:**
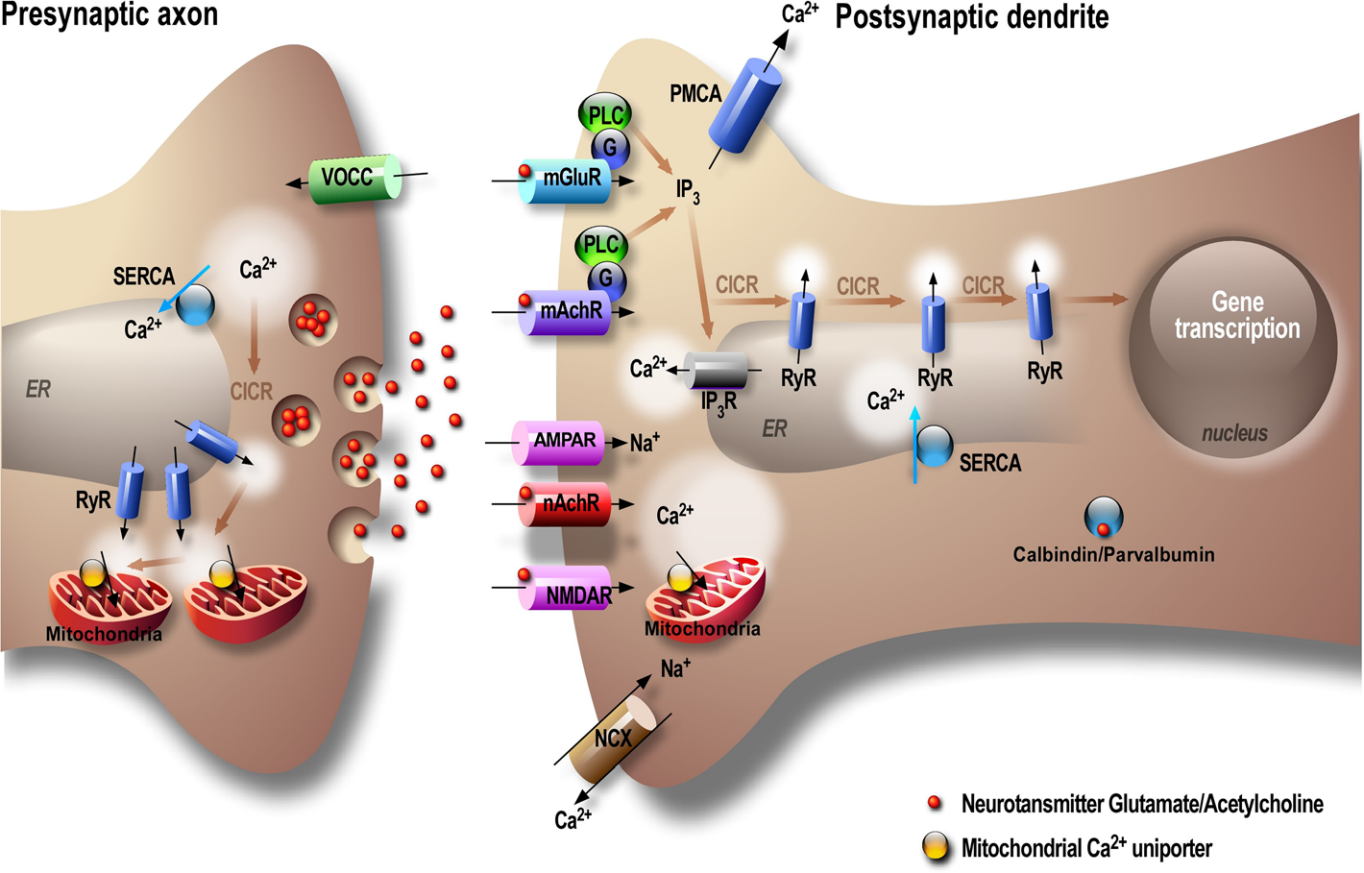
**Neuronal Ca**^**2+ **^**signaling.** Cytosolic [Ca^2+^]_i_ rises are the result of an influx across the plasma membrane via voltage-gated Ca^2+^channels (VGCCs), ionotropic glutamate receptors (N-Methyl-D-Aspartic acid receptors, NMDARs; and alpha-amino-3-hydroxyl-5-methyl-4-isoxazole-propionate, AMPARs), and the release from the ER through the inositol 1,4,5-trisphosphate (IP_3_R) and the ryanodine (RyR) receptors. Intraneuronal Ca^2+^ compartmentalization, is also maintained by the activity of Ca^2+^-binding buffering proteins (e.g., calbindin and parvalbumin), and regulated within signaling microdomains which involve, ATP-dependent Ca^2+^ pumps SERCA (Sarco-Endoplasmic Reticulum Ca^2+^ ATPase) accumulating Ca^2+^ from the cytosol to ER, and the sodium-Ca^2+^ exchanger (Na^+^/Ca^2+^), which act together with PMCA (Plasma Membrane Ca^2+^ ATPase) to restore [Ca^2+^]_i_ back to resting levels by extruding Ca^2+^ from the cytosol to the extracellular space. Although much of the Ca^2+^ entry into neuron is predominantly mediated by plasma membrane channels, IP_3_R- and/or RyR-mediated Ca^2+^ release can be subsequently recruited via the phenomenon of Ca^2+^-induced Ca^2+^ release (CICR), a regenerative process in which Ca^2+^ enhances its own release from IP_3_R and RyR.

Calcium (Ca^2+^) is a ubiquitous signal transduction molecule. It plays a key role in the modulation of neuronal activity and is involved into a wide array of cellular signals regulating various critical processes, such as cell growth, differentiation, metabolism, exocytosis, and apoptosis [10]. In neurons, the elevation of cytosolic Ca^2+^ concentration ([Ca^2+^]_i_) triggers the release of neurotransmitter at synaptic junctions and contributes to dendritic action potential, regulates the activity-dependent changes in gene expression, as well as synaptic plasticity [11]. Cytosolic Ca^2+^ levels are kept in a very low range (≈100 nM) compared with the levels present in the extracellular space (≈2 mM) or inside intracellular stores (≈100-500 μM), where the endoplasmic reticulum (ER) represents the major dynamic Ca^2+^ intracellular pool [12]. Neuronal Ca^2+^ signaling implicates a complex interplay between Ca^2+^ entry through the plasma membrane and release from the ER (Figure 2). The ER is a continuous and highly motile network distributed throughout the neuron within dendrites and dendritic spines, axons and presynaptic nerve terminals, as well as in growth cones [13] and supports diverse functions within each of these cellular compartments [14]. Thus, in dendrites, ER Ca^2+^ release is involved in modulating postsynaptic responses and synaptic plasticity [15]; in axon terminals, it is involved in vesicle fusion and neurotransmitter release [16]; in the soma, it is coupled to the activation of Ca^2+^-sensitive signaling pathways such as kinase and phosphatase activities [11]; and in the perinuclear space, it can trigger gene transcription [17]. Ca^2+^ mobilization from the ER is also important in growth cone activity involved in the formation of new connections and/or the strengthening of preexisting connections that occur during learning and memory in the adult brain [18].

The hypothesis that Ca^2+^ homeostasis perturbation could play a pivotal role in cascading events to AD was introduced more than 20 years ago [19]. Data obtained from experiments on dissociated cells, brains slices, and more recently and importantly on live AD animal models confirmed this hypothesis [20-23]. Data are now converging to demonstrate the important role of ER Ca^2+^ deregulation in AD [24,25]. In this review, we will specifically highlight how primary alterations of the expression and function of the ryanodine receptor (RyR) Ca^2+^ channels may play a key role in the setting of histopathological lesions, alteration of synaptic plasticity, and learning and memory deficits that are associated with the late stages of the disease and how these receptors may interplay with secretases expression and function and βAPP catabolites.

## Review

### RyRs expression and structure

RyRs are a family of three known mammalian isoforms: RyR1, RyR2, and RyR3 which are classified as “skeketal muscle”, “heart” and “brain” types, respectively with respect to their major tissue distribution although all isoforms can be found in the brain. Thus, RyR1 is expressed at low levels in cerebellum and Purkinje cells. RyR2 is predominantly expressed in Purkinje cells of cerebellum and cerebral cortex, and in dentate gyrus of the hippocampus. RyR3 has been detected in hippocampal CA1 pyramidal cell layer, the basal ganglia, and olfactory bulbs [26].

RyRs are homotetramers with a total molecular mass of about 2 MDa. Each subunit of the receptor is a compound with a molecular mass of about 565 kDa, with the 4/5 of the channel comprising an huge N-terminal cytoplasmic domain that serves as a scaffold for channel regulators while the remaining domain is in the ER lumen [27].

### RyRs pharmacology and regulation

Ca^2+^ activates all RyRs at low nanomolar concentrations with RyR1 > RyR2 > RyR3 in term of sensitivity to cytosolic free [Ca^2+^] [28,29]. Caffeine and ryanodine are pharmacological modulators of RyRs. Caffeine freely diffuses through the plasma membrane and subsequent cell excitation can be directly monitored by its ability to quench the fluorescence of various Ca^2+^ sensors in a wavelength-independent manner [30]. All structures of neurons, from soma to dendrites and presynaptic terminals, respond to caffeine. Caffeine induces a [Ca^2+^]_i_ rise without a requirement for extracellular Ca^2+^, and the [Ca^2+^]_i_ elevation is not associated with plasmalemmal Ca^2+^ movements. Caffeine-evoked [Ca^2+^]_i_ elevations are sensitive to pharmacological modulators interacting with RyRs or with SERCA pumps. Thus, these [Ca^2+^]_i_ responses are blocked by ryanodine, ruthenium red, and procaine and disappear after inhibition of SERCA-dependent ER Ca^2+^ uptake with thapsigargin (TG) or cyclopiazonic acid (CPA) [30]. Caffeine-induced Ca^2+^ release that is sensitive to TG and ryanodine has even been observed in individual spines of cultured hippocampal neurons, which are rich in RyR [31]. Ryanodine locks the RyR channel to an “open state” at low concentrations (<10 nM) and to “closed state” at higher concentrations (>100 μM) [32]. The activity of RyRs is also inhibited by mM concentrations of Mg^2+^ and μM concentrations of ruthenium red [32-34]. Dantrolene is well known inhibitor of RyRs. It was first characterized as a skeletal muscle relaxant [35], and is widely used in anesthesiology practice and in treating malignant hyperthermia (MH) [36]. Dantrolene was described to be bind to amino acids 590–609 of RyR1 isoform, which presumably stabilizes the channel protein, thus providing evidence for a direct action of dantrolene on RyRs [37,38]. This stabilizing effect inhibits aberrant activation of the channel and prevents excessive Ca^2+^ release from intracellular stores. Different papers also tend to show that dantrolene binds to the corresponding sequence (amino acids 601–620) of RyR2 [39,40]. Recent evidence has suggested that dantrolene may ameliorate abnormal RyR2-mediated Ca^2+^ release associated with heart failure [41-43]. It is worth to mention that the specificity of dantrolene towards RYRs has been debated in a recent review [23], since azumolene, an equipotent dantrolene analog, inhibits a component of SOCE coupled to activation of RyR1 by caffeine and ryanodine [23,44].

RyRs are also directly or indirectly modulated by other channels and kinases [45]. Calmodulin (CaM), a 17 kDa Ca^2+^ binding protein, binds to RyR1 at Cys3635 and to the corresponding region on RyR2, 3578–3603. CaM can either activate or inhibit RyR1 depending on Ca^2+^concentrations but appears to preferentially inhibit RyR2 [45,46]. The 12 and 12.6 kDa FK506 binding proteins (FKBP12 and FKBP12.6) known also as Calstabin1 and Calstabin2 bind to, and stabilize the closed state of RyR1 and RyR2, respectively, and are essential for coupled gating of RyR channels [47]. Ca^2+^/calmodulin-dependent protein kinase II (CaMKII) associates with, phosphorylates, and regulates the activity of RyR in the heart and skeletal muscle [48]. RyRs may also be regulated by Sorcin, a cytosolic Ca^2+^-binding protein [49], and by Calsequestrin (CSQ), a low affinity, high capacity luminal Ca^2+^ buffering protein [50]. Different studies reported that mutations in RyR or alterations in the post-translational modifications of RyRs (i.e. hyper-phosphorylation, -oxidation, and -nitrosylation) can shift RyRs from a finely regulated state to an unregulated Ca^2+^ leak channel. RyR “leaky” channels alter cell physiology and are associated with different pathological states in muscle and non-muscle cells [51-55].

### Overview of Ca^2+^ dysregulation in AD

Alteration of Ca^2+^ signals in AD were largely linked to the “amyloid hypothesis” where the modification of the nature, levels, biophysical properties and subcellular localization of Aβ peptides in various areas of the brain is believed to contribute to a molecular dysfunction culminating in neuronal death and dementia. A large number of studies also revealed the potent implication of PS as major contributor to Ca^2+^ deregulation in AD. The data listed below summarize the major findings obtained in this field:

I. In vivo imaging of Ca^2+^ transients in APP/PS1 (APP_swe_/PS1-ΔE9) mice model revealed that about 20% of transgenic mice dendrites and axons harbor moderate to severe [Ca^2+^] elevation than wild type mice, and that the severity of Ca^2+^ overload correlates with the structural integrity of the dendrite or axon. It was suggested that [Ca^2+^] elevation may depend on the Aβ accumulation since Ca^2+^ transients were not observed in animals without cortical plaques (i.e. young APP_swe_ and PS1 mutant mice) [56]. Another group reported a more complex alteration of Ca^2+^ transients in a different AD mice model (APP_swe_/PS1_G384A_) by showing a spatial distribution of silent neurons (decreased Ca^2+^ signals) (29%) and hyperactive neurons (increased Ca^2+^ signals) (21%) in transgenic mice brain as compared to wild type mice brain. In accordance with the paper by Kuchibhotla et al., they also proposed that the neurons hyperactivity occur near Aβ plaques [57].

II. Exogenous application of synthetic Aβ peptides or oligomeric agregates leads to elevated levels of [Ca^2+^]_i_[58,59]. Aβ-mediated changes in [Ca^2+^]_i_ occur likely through Aβ ion channels incorporation in the cell membrane, changes in membrane permeability, and phosphatidylserine asymmetry [60,61]. Actually, in addition to promoting influx of extracellular Ca^2+^[62], Aβ oligomers potently evokes Ca^2+^ signals through its release from the ER [58,63-65]. Another mechanism underlying Aβ-mediated elevated Ca^2+^ entry was recently revealed by renner et al. showing that soluble Aβ oligomers accumulation at the synapse recruited mGluR5 thus elevating intracellular Ca^2+^[66]. It is important to emphasize that the level of exogenously applied Aβ, used in these studies, is orders of magnitude above physiological levels. Thus, the relevance of Aβ-mediated Ca^2+^ signaling deregulation is provided in experiments using *in vitro* and *in vivo* AD models overproducing endogenously Aβ (see details in item IV).

III. Mutations of PS1 and PS2 had a significant impact on Ca^2+^ signaling in AD models. Actually, PS may directly alter ER Ca^2+^ signaling and affect activity and/or expression of many proteins involved in ER Ca^2+^ signaling deregulation in AD. Several studies showed that PS mutations induce exacerbated IP_3_R- and RyR- mediated Ca^2+^ release [67-72], and alter the function of the SERCA pump [73]. This has been documented in fibroblasts isolated from FAD patients, in cellular systems expressing wild type and mutated PS and in hippocampal and cortical neurons of AD mice [67,68,70-72]. PS were also shown to support ER Ca^2+^ leakage [74,75], likely through their function as low conductance, passive ER Ca^2+^ leak channels, independently of their γ-secretase activity [74,76-79]. Even if PS-mediated ER Ca^2+^ leak was recently debated [80,81], recent data obtained by other laboratories and using different systems tend now to confirm the leak function of PS [82,83].

IV. Other studies have identified βAPP-mediated changes to ER Ca^2+^ signaling related to Amyloidogenic processing of βAPP. Decreased production of sAPPα (soluble APPα fragment: derived from non-amyloidogenec processing of βAPP) (Figure 1), was shown to activate K^+^ channels [84]. The transcription regulatory factor AICD (APP intra-cellular domain: derived from both amyloidogenic and non-amyloidogenic βAPP processing) (Figure 1) may affects Ca^2+^ hormeostasis by regulating the expression of genes involved in Ca^2+^ homeostasis [85-87], namely the transient receptor potential cation channel subfamily C member 5 (TRPC5), a component of receptor-activated nonselective Ca^2+^ permeant cation channel [88]. Additional studies support the physiological role of βAPP in Ca^2+^ homeostasis by demonstrating that βAPP downregulation enhances both Ca^2+^ content of the ER and acidic stores and the dynamics of store operated Ca^2+^ channel activity [89]. As for PS, βAPP FAD mutations were also shown to alter Ca^2+^ signals. It has been documented that fibroblasts from AD patients harboring the Swedish double mutation (βAPP_swe_: βAPP_K670N/M671L_) showed reduced bombesin-induced intracellular Ca^2+^ elevations compared to controls while all other pools of Ca^2+^ were unaffected [90]. Primary cortical neurons from TgCRND8 mice carrying combined βAPP_swe_ and Indiana (βAPP_V717F_) mutations show elevated ER release of Ca^2+^[91]. In accordance with these findings, we recently reported a global alteration of Ca^2+^ homeostasis in human neuroblastoma SH-SY5Y cells overexpressing human wild type βAPP or βAPP_swe_. This Ca^2+^ alteration is manifested by an increase in cytosolic Ca^2+^ signals associated to enhanced ER Ca^2+^ passive leakage, and large IP_3_R- and RyR-mediated Ca^2+^ release as compared to control cells, and to increased VGCC permeability to Ca^2+^[92].

### A focus on Ryanodine Receptors-mediated Ca^2+^ signals deregulation in AD

Several studies addressed the role of RyR-mediated Ca^2+^ disruptions in AD models (Table 1). It was shown that the RyR blocker dantrolene reversed carbachol-induced elevation of Ca^2+^ release in human neuroblastoma SH-SY5Y cells expressing PS1 mutants (PS1_M146V_, and PS1_L250S_) [93]. Accordingly, it was also reported that the RyR agonist caffeine evoked larger Ca^2+^ liberation in primary cultured neurons derived from the triple transgenic mice model 3xTg-AD (knock in for the mutated PS1_M146V_, and overexpressing mutated βAPP and microtubule-associated tau protein (PS1_M146V_/APP_swe_/tau_P30IL_)), and the transgenic knock in mice model expressing mutated PS1 (PS1_M146V_) [94]. Increased RyR channel function was further confirmed in PC12 cells expressing wild type PS1, PS1_L286V_, PS1_M146V_ or PS2_N141L_ mutants [95,96]. Interestingly, RyR-mediated ER Ca^2+^ homeostasis deregulation in AD was supported by the finding showing that exacerbated IP_3_R-evoked Ca^2+^ signals in the PS1_M146V_- and the 3xTg-AD-derived neurons occur through increased RyR-mediated CICR (Ca^2+^-induced Ca^2+^ release) (Figure 2) [72]. Studies by the group of Bezprozvanny postulated that the large RyR-mediated Ca^2+^ release observed in the 3xTg-AD-derived neurons is likely associated to the impairment of PS Ca^2+^ leak channel function and to increased ER Ca^2+^ pool [78]. The emerging hypothesis form these studies is that RyR function alteration may be intimately linked to PS. However, experiments using the PScDKO mice model (PS1 and PS2 conditioned double knockout mice) lead to controversial conclusions [78,97]. While the group of Bezprozvanny showed that primary neurons derived from PScDKO mice harbor increased ER Ca^2+^ pool and increased RyR-mediated Ca^2+^ signals [78], a recent study by Wu et al. showed that neurons derived from the same study model did not harbor alteration in ER Ca^2+^ content and rather display reduced RyR agonist-induced Ca^2+^ release from the ER and RyR-mediated synaptic responses [97]. Furthermore, the authors demonstrated that knockdown of RyR expression in wild-type hippocampal neurons mimics the defects observed for Ca^2+^ homeostasis and presynaptic function in PScDKO neurons [97]. These data further support previous results demonstrating a physiological role of PS in synaptic plasticity [98,99], and that inhibition of RyR function mimics and occludes the effects of PS inactivation and intracellular Ca^2+^ homeostasis and synaptic dysfunction [99].

**Table 1:** RyR-mediated calcium deregulation in AD

**AD models**	**Study systems**	**RyR-mediated [Ca**^**2+**^**] dysregulations**	**References**
PS1_M146V_ and PS1_L250S_	SH-SY5Y cells	Dantrolene blocked ↑ carbachol-induced [Ca^2+^] signals (vs. wild type PS1)	[93]
3xTgAD mice PS1_M146V_ KI Tg mice	Primary cortical neurons	↑ caffeine-induced [Ca^2+^] signals (vs. wt neurons)	[94]
PS1_WT,_ PS1_L286V,_ PS1 _M146V,_ PS2 _N141L_	PC12 cells	↑ caffeine-induced [Ca^2+^] signals (vs. vector transfected cells)	[95,96]
APP_wt_ and APP_swe_	SH-SY5Y cells	↑ caffeine-induced [Ca^2+^] signals (vs. vector transfected cells)	[92]
Tg2576 mice	hippocampal primary neurons	↑ caffeine-induced [Ca^2+^] signals (vs. wt neurons)	[92]
PS _M146V_ KI Tg and 3xTgAD mice	Acute brain slice preparation (6w, 6 mo and 1.5 Y) (*)	↑ caffeine-induced [Ca^2+^] signals	[72,100]
		Dantrolene reduced the IP_3_-evoked Ca^2+^ responses (vs. wt-derived brain slices)	
Extracellular Aβ42 application	Primary Cortical neurons	siRyR-3 blocked increased ryanodine- and glutamate-induced [Ca^2+^] signals upon Aβ42 application (vs. Aβ42 non-treated neurons)	[91]
PScDKO Tg and 3xTgAD mice	Primary hippocampal neurons	↑ caffeine-induced [Ca^2+^] signals (vs. wt-Tg neurons)	[78]
		↑ ER Ca^2+^ pool (vs. wt-Tg neurons)	
PScDKO Tg mice	Primary hippocampal neurons	↔ ER Ca^2+^ pool	[97]
		↓ caffeine-induced [Ca^2+^] signals (vs. wt neurons)	
PS2_N141L_ Tg and PS2_N141L_/APP_swe_ mice	Primary neuronal cultures and acute brain slice preparation	↓ ER [Ca^2+^]	[101]
		↓ IP_3_-generating Ca^2+^ responses	
		↑ caffeine-induced [Ca^2+^] signals (vs. wt-Tg neurons)	
PS1_M146V_KI Tg and PS1_M146V_/APP_swe_ Tg mice	Acute brain slice preparation	↑ caffeine-induced [Ca2+] signals	[102]
		RyR blockade prevents NMDA Ca^2+^ response (vs. wt-derived brain slices)	

*Abbreviations*: [*Ca*^*2+*^] calcium signals are depicted as ↑ (increased), ↓ (reduced), or ↔ (unchanged) as compared to respective controls, (*) study was performed on acute brain slice preparation isolated from transgenic mice at different ages (6 weeks (w), 6 months (mo) and 1.5 years (Y), *vs* versus, *KI* knock in, *Tg* Transgenic, *wt* Wild type, PScKO Tg mice are conditionally double knock out for PS1 and PS2, Tg2576 mice express the βAPP harboring Swedish double mutation (*APP*_*K670N*_*/*_*M671L*_), 3xTgAD mice are generated from the PS1_M146V_ KI mouse overexpressing APP_swe_ and Tau_P130L_.

These studies may outline a possible regulation between RyR and PS towards ER Ca^2+^ homeostasis. However, the mechanisms underlying this regulation are still unraveled and the basis of the contradictory results in PSDKO cells is not clear.

Another question emerging in “AD Ca^2+^ hypothesis” is the real impact of PSs versus PSs-mediated βAPP processing and Aβ peptides production in Ca^2+^ disturbances in AD models. To answer this question, in a recent study, Kipanyula and colleagues used two transgenic mice models carrying the FAD-linked PS2_N141L_ mutation either alone or in the presence of βAPP_swe_ mutation (PS2-APP) [101]. They reported in both PS2_N141L_ and PS2-APP transgenic neurons a similar reduction in ER Ca^2+^ content and decreased response to IP_3_-generating agonists, albeit increased Ca^2+^ release induced by caffeine and increased Ca^2+^ excitability. Further experiments lead authors to postulate that enhanced response to caffeine in both models resulted from the increased level of RyRs observed in brains and cultured neurons derived from both transgenic mice as compared to wild-type mice [101]. The comparative analyses of both transgenic mice models lead authors to hypothesize that Ca^2+^ stores deregulation depend directly on the mutant PS2 itself and not on PS2-dependent APP processing or total Aβ levels (i.e. larger Aβ level was detected in PS2-APP mice as compared to PS2 mice) [101]. However, it may be argued that the Aβ increase observed in PS2 transgenic mice models may be sufficient to cause the Ca^2+^ alterations and that the additional larger Aβ rise observed in the PS2-APP mice have no additional effect. In this scenario, RyR dysfunction in PS2 and PS2-APP mice may also likely and predominantly dependent on a direct interaction of PS2 mutant with RyR.

Importantly, the real impact of Aβ and of βAPP overexpression and mutation on ER Ca^2+^ signaling and particularly on RyR dysfunction was revealed in AD-related study models independently from PS mutation or overexpression [64,86,91,92,103,104]. Regarding βAPP overexpression and mutation, it has been documented that primary cortical neurons isolated from TgCRND8 mice (described above) display elevated RyR-mediated Ca^2+^ release, while global Ca^2+^ handling remained unaffected [91]. Accordingly, we recently highlighted a fundamental role of increased RyR-induced Ca^2+^ release in SH-SY5Y neuroblastoma cell line stably overexpressing either wild-type or mutated human βAPP (APP_695_ or APP_swe_ respectively), and in primary neurons from APP_swe_-expressing mice (Tg2576) [92]. Of most interest, it was also reported that exogenous Aβ oligomers may stimulate RyR-mediated Ca^2+^ release in wild type hippocampal neurons [105], and that application of soluble Aβ caused a marked increase in RyR activity, resulting in a 10-fold increase in channel open probability which was blocked by RyR antagonist ruthenium red [106].

### Ryanodine Receptors expression in AD

The alteration of RyR expression in AD-affected brains was first described in 1999 by Kelliher et al. [107], who showed that [^3^H] ryanodine binding (indicative of RyR expression protein) is elevated in hippocampal regions (subiculum, CA2 and CA1) of human post-mortem tissue at early stages of the disease, i.e. prior to extensive neurodegeneration and overt Aβ plaque deposition [107]. Recently another paper reported elevated RyR2 mRNAs levels early in mild cognitive impairment derived brains [108]. Accordingly, altered RyR2 expression was recently reported in a preliminary study of the whole-genome expression profile of sporadic and monogenic early-onset AD [109] (Table 2).

**Table 2:** RyR expression in Alzheimer disease

**AD samples/models**	**Brain regions/study systems**	AD stage (*)	RyR expression (**)	**References**
Human AD post-mortem brains	Hippocampal regions (Subiculum, CA1, CA2)	Early stages (I-II)	↑ [^3^H] RyR binding	[107]
Human AD post-mortem brains	Hippocampal regions (Subiculum, CA1, CA2, CA3, and CA4)	Late stages (V-VI)	↔ [^3^H] RyR binding	[107]
			↓RyR2 mRNA	
PS1 _L285V_, PS1_M146V_ mutations	PC12 cells		↑ RyR3 mRNA and protein	[95]
PS1_M146V_ KI Tg mice	Primary neurons			
Human AD post-mortem brains	mid frontal cortex		↓RyR2-3 mRNA splice variants	[108]
MCI post-mortem brains	mid temporal and mid frontal cortex		↑RyR2 mRNA; ↔ RyR3 mRNA	[108]
	mid frontal cortex		↓RyR2 mRNA splice variant;	
	mid temporal cortex		↑RyR2 mRNA splice variant	
Extracellular Aβ42 application	Primary cortical neurons		↑RyR3 mRNA;	[91]
			↔ RyR1-2 mRNA	
CRDN8 Tg mice	Primary cortical neurons	4-4.5 (mo)	↑RyR3 mRNA	[91]
	Whole brains		↑RyR3 protein	
3xTgAD mice	Primary hippocampal neurons		↑RyR protein	[78]
PS1_M146V_ KI Tg mice 3xTgAD mice	Whole brains	6 (w)	↑RyR protein	[72,110]
		6 (mo)	↔ RyR protein	
		1.5 (Y)	↑RyR protein	
3xTgAD mice	Hippocampus	6-8 (w)	↑RyR2 mRNA;	[94,100]
			↔ RyR1-3 mRNA	
Tg2576 mice	Cortex	15-18 (mo)	↑RyR2 mRNA;	[92]
			↔ RyR1-3 mRNA	
			↑RyR proteins	
APP_Wt_ and APP_swe_ mutation	SH-SY5Y cells		↑RyR1-2-3 mRNA	[92]
			↑RyR proteins	
PScDKO Tg mice	Hippocampus	2 (mo)	↓ RyR proteins	[97]
			↓ [^3^H] RyR binding	
			↔ RyR mRNA	
Subletal Aβ oligomers	Primary hippocampal neurons		↓ RyR 2–3 mRNA	[105]
			↓ RyR 2 protein	
APP_swe_PS1_L166P_ Tg mice	Hippocampus	3 and 6 (mo)	↑RyR 2–3 protein	[111]

*Abbreviations*: (*) AD stage is defined following BRAAk’s staging for human AD post-mortem brains, and is referred in weeks (W), months (mo) or years (Y) in AD mice models, (**) RyR expression (mRNA or protein level) is depicted as ↑ (increased), ↓ (reduced), or ↔ (unchanged) as compared to respective controls, *PS* presenilin, *MCI* mild cognitive impairment, *KI* knock in, *Tg* transgenic, *Wt* wild type, CRDN8 Tg mice express human APP with a double mutation (APP_K670N_/_M671L_ + APP_V717F_), PScKO Tg mice are conditionally double knock out for PS1 and PS2, Tg2576 mice express the Swedish double mutation (APP695_K670N_/_M671L_), 3xTgAD mice are generated from the PS1_M146V_KI mouse overexpressing APP_swe_ and Tau_P130L_.

Interestingly, alteration of RyR expression was reported in different AD study models and was shown to be linked to Aβ and/or overexpression of PS mutants. Thus, increased expression of RyR3 but not RyR1 or RyR2 was observed in cortical neurons isolated from C57Bl6 mice upon extracellular Aβ42 application [91], and in cortical neurons and brain tissue from TgCRND8 mice [91]. We recently showed that RyR protein and mRNAs levels are increased in neuroblastoma SH-SY5Y cells overexpressing wild type βAPP or βAPP_swe_ and in Tg2576 mice as compared to respective control mice [92] (Table 2).

Increased RyR expression was also largely reported in AD models where PS is overexpressed or mutated. Elevated RyR mRNAs and protein was first reported in *in vitro* models expressing PS1 mutants [95]. Importantly, RyR expression increases throughout the lifetime of the PS1_M146V_, and the 3xTg-AD transgenic mice [72,94,100]. Recent findings of Liu et al. further support these observations by showing increased expression of both RyR2 and RyR3 in APP_swe_PS1_L166P_ transgenic mice [111]. It was proposed that the induction of RyR expression is a compensatory event linked to the loss of PS leak function [78]. This hypothesis was recently debated in a study using PSDKO mice model, where authors revealed that the absence of PS, on the contrary, triggers an hippocampal reduction of RyR protein levels [97]. The basis of the contradictory results about RyR expression in AD models may be linked to a variable regulation of RYR along AD pathology development and between brain areas. As a matter of fact, reduction of RyR2 and RyR3 mRNA levels and RyR2 protein expression was observed upon treatment with sublethal concentrations of Aβ oligomers [112]. Kelliher, M. et al. [107] also reported a complex regulation of RyR expression in human AD brains where RyR expression was shown to be elevated in hippocampal regions in cases with early neurofibrillary pathology and reduced in the subiculum, and CA1-CA4 regions of the late stages [107] (Table 2).

The molecular mechanisms that could underlie the regulation of RyR expression in AD are still unknown. ER stress is induced during AD, and has been indirectly implicated as a mediator of Aβ neurotoxicity [113]. In this context, it was demonstrated that Aβ triggered the activation of the ER stress response factor X-box binding protein 1 (XBP1), thereby yielding its transcriptionally spliced active form XBP1s. XBP1s showed neuroprotective activity towards Aβ oligomers through a reduction of cytosolic Ca^2+^ and of the expression of RyR3 [114]. Human and murine RyR3 contains multiple XBP1s binding sites [114]. It is however still unclear whether the regulation of RyR3 by XBP1 is direct or indirect. Additional work is necessary to demonstrate these observations.

### The molecular link between Ryanodine Receptors and presenilins

In many cases, the adverse effects of PS mutations on Ca^2+^ homeostasis were associated to RyR channels activity alteration. However, the molecular mechanism underlying this regulation is not fully understood. Nevertheless, RyRs were shown to co-localize with PSs at the ER membrane [96]. Co-immunoprecipitation experiments also unraveled a physical interaction between PS1-2 and RyR2 [95,96,115]. The laboratory of Peter Koulen further investigated the possible physical interaction of PS with RyR and studied *in vitro* the impact of such interaction on RyR channel activity. In two different studies, they demonstrated that PS1 and PS2 N-termini fragments strongly increased both mean currents and open probability of single brain RyR channels [116,117]. They proposed that PS1 NTF (1–82) and PS2 NTF (1–87) may interact with the cytoplasmic side of RyR and allosterically potentiates RyRs in a Ca^2+^-dependent manner [116,117]. Another group demonstrated the molecular interaction of the large hydrophylic C-terminal region of PS2 with sorcin, a modulator of RYR channel, in human derived neuronal cell line and in brain tissues. Their data also suggested that PS2/sorcin interaction is potentiated by Ca^2+^[118]. This observation was further confirmed in another cellular system where PS2, sorcin, and RyR2 were shown to physically interact (in a Ca^2+^ dependent manner) in both HEK-293 cells overexpressing these proteins and in mouse hearts [115].

All together this set of data demonstrates that PS may play an important role in RyR channel activity directly through the interaction of PS with RyR and indirectly through the interaction of PS with RyR modulators. However, the implication of such RyR channel function regulation by PS for AD remain to be elucidated (i.e. study of PS1-2 mutants physical interaction with RyR and the potential effect of such interactions on RyR channel activity).

### Post-translational Ryanodine Receptors modification in AD: just a hypothesis

Enhanced RyR-mediated ER Ca^2+^ depletion may be linked to pathophysiological post-translational modifications in the macromolecular complex containing RyR1 or RyR2 resulting in “leaky channels” [45]. Interestingly, post-translational modifications of RyR2 were reported in cerebral ischemia [119] where endogenous RyRs undergo S-nitrosylation and S-gluthathionylation processes that resulted in high activity channels and ultimately lead to cortical neuronal death [119]. Disrupting function of FKBP1b, a RYR2 stabilizer, was recently shown to alter Ca^2+^ homeostasis in hippocampal neurons and to trigger the aging phenotype of Ca^2+^ deregulation in young animals (i.e. enhanced ryanodine sensitive AHP “after hyperpolarization” signals, and increased CICR from RyR) [120]. Recently, Liu et al. described the contribution of “leaky” RyR2 to stress-related memory impairments [55]. Through a series of biochemical, neurophysiological, and behavioral assays, the study demonstrated that chronic stress can affect RyR2 function through PKA hyperphosphorylation, oxidation, and nitrosylation leading to the physical dissociation of FKBP12.6/1b (calstabin2) and RyR2, thereby inducing Ca^2+^ leak from RyRs and cognitive dysfunction [55].

Post-translational modifications of RyRs in AD have not been reported yet. However, AD brains manifest excessive generation of reactive nitrogen (RNS) and oxygen (ROS) species, contributing to neuronal cell injury and death via a series of redox reactions [121-123]. In addition, PKA activation was shown to be implicated in AD through the regulation of βAPP processing [124-126], Aβ-mediated cell death *in vitro* and *in vivo*[127,128], and oxidative stress. It is intriguing to note that RyR hyperphosphorylation is largely linked PKA activation [55,129]. Interestingly, a microarray analysis performed on CA1 hippocampal gray matter of Alzheimer and control subjects revealed the correlation of a down regulation of the expression of RyR stabilizers namely, FKBP1a with incipient AD, suggesting an additional mechanism involved in RyR dysfunction in AD [130]. All over, these observations must stimulate the initiation of dedicated studies investigating the influence of RyR destabilization in the onset and/or progression of AD.

### Ryanodine Receptors and βAPP processing

It was reported that Ca^2+^ homeostasis may influence βAPP pathophysiological processing. Thus, Aβ production is enhanced by elevation of intracellular [Ca^2+^]_i_[131,132] and RyR-mediated Ca^2+^ release [133], and is reduced in IP_3_R-deficient lines [67]. Of particular interest, we showed that dantrolene-induced lowering of RyR-mediated Ca^2+^ release leads to the reduction of βAPP cleavage by β- and γ-secretases and decreases both intracellular and extracellular Aβ load in wild type βAPP- or βAPP_swe_- overexpressing neuroblastoma cells as well as in primary cultured neurons derived from Tg2576 mice brain. We also demonstrated that this Aβ reduction could be accounted for by decreased Thr-668-dependent βAPP phosphorylation and lowered β- and γ-secretases activities [92]. Importantly, we and other laboratories showed that dantrolene diminishes Aβ load, and reduces Aβ-related histological lesions in three different AD mice models (Tg2576, 3xTg-AD and PS1_M146V_/APP_swe_), demonstrating that subchronic blockade of RyR activity may be beneficial in the context of AD [92,134,135]. On the contrary, long-term feeding of dantrolene was shown to increase Aβ load in APP_swe_PS1_L166P_ transgenic mice [78].

Discrepancies in the above-described data may rely on the kinetics of AD-like set-up and progression as well as the duration of RyR blockade specifically linked to various models examined. These puzzling results may also point out a possible dual protective/compensatory *versus* pathogenic role of RyR at different stages of the development of AD.

The recent paper by Bezprozvanny’s group sheds new lights on this complex dual role of RyR in the development of AD [111]. Authors used a genetic approach to modulate RyR expression along AD development in APP_swe_PS1_L166P_ mice and generated APPPS1xRyR3−/− mice as a study model. They demonstrate that the deletion of RyR3 in young APPPS1 mice elevates Aβ accumulation, and increases hippocampal neuronal network excitability thus accelerating AD pathology. In contrast, the deletion of RyR3 in older APPPS1 mice reduces Aβ plaques, and rescues the network excitability and the loss of mushroom spines [111].

All together, these studies suggest a complex dual role of RyR in AD pathology. RyR may function as a compensatory/protective actor at early disease stages, and acts as a pathogenic molecular determinant contributing to the setting of histopathological lesions and synaptic deficits observed at the late disease stages.

### Ryanodine Receptors and neurodegeneration

RyR-mediated ER Ca^2+^ release leads unequivocally to large cytosolic Ca^2+^ signals. Thus, it could be postulated that enhanced RyR-mediated Ca^2+^ release may be indirectly implicated in neuronal death through cytosolic Ca^2+^ overload. Indeed, pharmacological targeting of RyR by its specific inhibitor, dantrolene suggested that this receptor could play a direct role in neurodegeneration. Popsecu et al. showed that dantrolene protected neurons against kainic acid-induced apoptosis *in vitro* and *in vivo*[136]. Neuroprotective effect of dantrolene was also reported in cerebral ischemia [137,138], and in different neurodegenerative diseases such as Huntington’s disease [139,140], and spinocerebellar ataxia of types 2 and 3 [141,142]. Dantrolene was shown to reduce the glutamate-induced increases in intracellular [Ca^2+^], and protects against glutamate-induced neurotoxicity [143]. Complete block of glutamate toxicity by dantrolene was also observed in the absence of extracellular Ca^2+^, which indicates that Ca^2+^ release from intracellular stores is essential for the propagation of glutamate-induced neuronal damage [144].

Related to AD, intracellular Ca^2+^ levels were increased in cells expressing the human PS1_L286V_ mutation. Aβ induced cell death in these cells and dantrolene protected the cells against these deleterious effects [145]. Imaizumi et al. showed that treatment of rat cortical neurons with Aβ increased the expression DP5, a neuronal apoptosis-inducing gene [146]. Induction of DP5 gene expression was blocked by dantrolene suggesting that the induction of DP5 mRNA occurs downstream of the increase in cytosolic Ca^2+^ concentration caused by Aβ. Moreover, DP5 specifically interacts with Bcl-xl during neuronal apoptosis following exposure to Aβ, and its binding could impair the survival-promoting activities of Bcl-xl [146]. Accordingly, dantrolene treatment protected PC12 cells expressing PS2_N141L_ mutant from death induced by L-glutamate and Aβ toxic peptides [96]. The direct implication of RyR in neuronal death was also proposed by Supnet et al. who showed that suppression of RyR3 expression in TgCRND8 neurons, increased neuronal death [91,147], thus supporting a protective role of RyR in the late stages of AD pathogenesis, at least in this mice model. According to this, other studies highlighted a potential protective role of RyR in AD models. Thus, long-term pharmacological blockade of RYR with dantrolene in APP_swe_/PS_L166P_ mice resulted in the loss of synaptic markers, and neuronal atrophy in hippocampal and cortical regions [78].

Based on these results, one could assume that alteration of RyR-mediated Ca^2+^ signals along AD pathogenesis progression may shift cell behavior from a protective/adaptive response to a pro-apoptotic phenotype.

Dantrolene-mediated neuroprotection may accur via the modulation of Ca^2+^-dependent proteases and kinases. Thus calpain, CaMKII, PKA, and MAPK that are all activated by cytosolic Ca^2+^ control the transcriptional activation of immediate early and memory genes [148,149]. It is noteworthy that the activated form of calpain2 is increased in neuritis and neurons at risk for developing neurofibrillary pathology and is extensively bound to neurofibrillary tangles in brain AD patients [150]. Interestingly, calpains inhibition was demonstrated to improve memory and synaptic transmission in AD models [149]. Calcineurin (Ca^2+^/calmodulin-dependent serine/threonine phosphatase) induces endocytosis of NMDA receptors, reduces synaptic currents and activates propoptotic molecules such as BAD [151]. Remarkably, Dinely et al. also reported that calcineurin is upregulated in the brain of Tg2576 mice model [152], and that the calcineurin inhibitor FK506 prevents the loss of mitochondrial potential induced by Aβ by preventing cytochrome *C* release from mitochondria [153].

### Ryanodine Receptors-mediated synaptic dysfunction and learning and memory deficits in AD

#### Physiological role of RyR in synaptic function and memory formation and storage

Certain forms of Ca^2+^-dependent synaptic plasticity, including long-term potentiation (LTP) and long-term depression (LTD), are thought to underlie the cellular/molecular mechanisms of learning and memory [154]. High concentration spikes of Ca^2+^ activate LTP. Information placed in this temporary memory is uploaded and consolidated in more permanent memory stores during certain phases of sleep. During another phase of sleep, smaller elevation in Ca^2+^ activates the LTD.

Pre-synaptically, CICR waves through the RyR trigger neurotransmitter release that is detected as a temporary depolarization of postsynaptic membrane potential (miniature postsynaptic potentials) [155]. CICR evoked by voltage-dependent Ca^2+^ entry can mobilize neurotransmitter vesicles from the reserve pool to the readily releasable pool and thus facilitate subsequent vesicle release [156]. This Ca^2+^-dependent release has implications for short-term forms of presynaptic plasticity known as paired pulse facilitation (PPF) [102,155]. Post-tetanic potentiation (PTP) (another form of presynaptic plasticity that occurs following a high frequency stimulus) is also thought to involve residual Ca^2+^ elevation that results from RyR-mediated Ca^2+^ release [155,157].

Post-synaptically, ER Ca^2+^ is involved in long- and short-term plasticity. Long-term changes in synaptic efficacy and plasticity depend on the combination of channels recruited in dendritic spines [158]. For example, NMDAR mediated-Ca^2+^ entry into spines and dendrites is essential but not sufficient for the induction of LTP [156,159]. ER Ca^2+^ stores can amplify the initial NMDAR-mediated signal and determine the polarity as well as input specificity to activate downstream cascades necessary to encode LTP or LTD [159]. As a matter of fact, blocking IP_3_R leads to a switch of LTD to LTP and elimination of heterosynaptic LTD, whereas blocking RyR eliminates both LTP and homosynaptic LTD occurring at synapses that are activated, normally at low frequencies [160-163]. Finally, type 3 RyR knockout mice were shown to harbor enhanced LTP and reduced LTD [164,165].

Ca^2+^ partly regulates activity-dependent membrane excitability-sensitive K^+^ channels, such as SK channel, which contribute to the medium after-hyperpolarization (known also as refractory period, where in neuron membrane potential falls below the resting membrane potential). This current underlies spike-frequency adaptation, a phenomenon wherein accumulating Ca^2+^ entering through spiking activity reaches sufficient levels to activate hyperpolarizing K^+^ currents and transiently suppress membrane excitability. SK channels are largely activated by VGCC. However, IP_3_R- and RyR-mediated Ca^2+^ release were also shown to activate these channels and modify spiking patterns, thereby influencing local circuit activity [166].

#### RyR-mediated synaptic dysfunction in AD

Alteration of both RyR expression and function over time could have a significant effect on synaptic function that may contribute to cognitive decline. At the level of neuronal shape, augmented cytosolic Ca^2+^ leads to a loss of Ca^2+^ compartmentalization in dendritic spines and to a distortion of neurite morphologies mediated by activation of Ca^2+^-dependent phosphatase calcineurin [56]. Focusing on the role of RyR-mediated Ca^2+^ deregulation, Stutzmann’s group provided a large amount of data supporting the major role of RyR expression and function deregulation in synaptic abnormalities in AD mice models [72,100,135,165-168]. They first revealed that IP_3_-evoked membrane hyperpolarization is driven by Ca^2+^ liberation through RyR and enhanced coupling efficiency between RyR and Ca^2+^-activated K^+^ conductance [72,165]. Increased RyR-evoked Ca^2+^ release was shown to occur within synapse-dense regions of CA1 pyramidal neurons of young 3xTg-AD mice and the double transgenic mice co-expressing mutated PS1 and βAPP (PS1_M146V_/APP_swe_) and significantly increases the amplitude of spontaneous postsynaptic potentials and the frequency of events responses in 3xTg-AD as compared to non-transgenic neurons [100,166]. The obtained data also demonstrate that both presynaptic and postsynaptic RyR-sensitive Ca^2+^ stores contribute to synaptic transmission and plasticity in 3xTg-AD but not in non-transgenic mice [100,166]. Interestingly, these signaling changes are present before Aβ formation, tau deposits, or memory deficits thus revealing RyR function alteration may represent an early pathogenic process of AD [100,166].

Recently, by using young 3xTg-AD mice, the same laboratory showed that under control conditions, basal synaptic transmission, PPF and LTP appear similar to the non-transgenic mice. However when RyRs are blocked and enhanced CICR effect was suppressed, the AD neurons demonstrated enhanced basal synaptic transmission and altered short and long term plasticity. This may suggest that RyR-mediated Ca^2+^ signals have a prominent inhibitory effect in basal synaptic transmission and presynaptic neurotransmitter release in the AD mice [135,168]. These data were further confirmed by showing that sub-chronic stabilization of ER Ca^2+^ signaling earlier in the disease process has beneficial effects on synaptic transmission and plasticity abnormalities in presymptomatic 3xTg-AD mice and adult PS1_M146V/_APP_swe_ double transgenic mouse model, while having little effect in non-transgenic controls [135,168].

#### RyR-mediated learning and memory decline in AD

Some evidences suggest that RyR expression levels may have a direct role in behavior and cognitive trait. It was shown that nicotine administration upregulates RyR2 levels in brain areas that control cognitive and motivational systems (notably the cortex and ventral midbrain) [169]. Previous works have shown that RyR mRNA and protein levels in the hippocampus are upregulated in response to spatial learning tasks [170]. In agreement with the role of RyR in synaptic plasticity, it was reported that RyR may play a role in learning and memory. Thus, RyR3 knockout mice exhibit decreased social behavior [171], and memory retention is accompanied by increased expression of RyR2 mRNA in the hippocampus of water maze-trained rats as compared to swimming controls [172].

In the context of AD, we demonstrated that dantrolene reduced Aβ burden in Tg2576 mice *in vivo*. The influence of dantrolene on learning and memory decline was studied by means of two complementary tests: the Morris water maze (MWM), which examines spatial memory, and the novel object recognition (NOR) paradigm, which records recognition memory. Both tests revealed alterations of both learning and memory behavior in Tg2576 mice. Chronic treatment with dantrolene does not affect learning ability in wild type mice, but restores learning ability in Tg2576 mice (MWM test), and increased the object discrimination index when compared to vehicle-treated Tg2576 mice (NOR test) [92]. According to these data, Peng et al. showed that chronic treatment with dantrolene reverses memory decline in the 3xTg-AD by using the MWM test [134].

We showed that Tg2576 mice harbor a reduced level of PSD-95 (a component of the post-synaptic density membrane associated guanylate kinase (PSD-MAGUK) scaffolding proteins) [92]. It is well established that PSD-MAGUK indirectly regulates synaptic plasticity and memory through the control of the number and compartmentalization of both AMPA and NMDA glutamate receptors around the PSD (post-synaptic density) [173]. We hypothesized that excessive RyR-mediated Ca^2+^ release and subsequent increased Aβ load may have contributed to PSD-95 expression decline in Tg2576 mice which may have led directly or indirectly to learning and memory decline. The recently published paper by Liu et al. is in accordance with these findings since genetic depletion of RyR in old APPPS1 mice rescued neuronal network excitability [111].

## Conclusion

Obviously, this review reveals RyR as a key molecular determinant in “AD Ca^2+^ hypothesis”. It also highlights the molecular mechanisms that could influence RyR-mediated Ca^2+^ release in AD where PS and Aβ emerge as detrminant regulators of RyR expression and function alteration.

**Figure 3:**
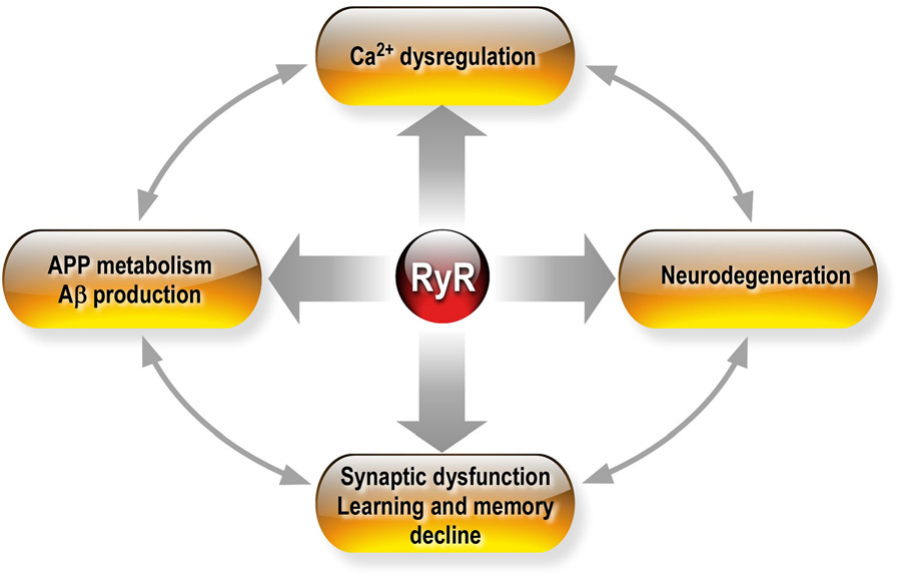
**Scheme of the implication of RyR expression and function alterations in AD.** RyR-mediated AD pathogenesis occurs through Ca^2+^ signaling dysregulation, the amplification of βAPP metabolism and Aβ peptide production, the control of neuronal death and degeneration, synaptic dysfunction, and learning and memory decline. The functional cross talk between these AD “pathological hallmarks” places RyR at the crossroads of AD pathogenesis.

Altered RyR levels have been described early in human AD cases, in mild cognitive impairment and in various AD models [107,108]. Accordingly, deregulation of RyR function was reported in diverse *in vitro* and *in vivo* AD study models. Data interpretation concerning some controversial results about RyR deregulation in AD must take in consideration the divergence of study systems i.e.: i) simple versus double or triple transgenic mice models; ii) primary cultures neurons and acute hippocampal slices versus neuronal derived cell lines or fibroblasts; and most importantly iii) the time course of “AD pathogenesis” development in each study model. We have also to consider that AD-associated neurodegeneration, synaptic dysfunction and cognitive decline are complex, inter-regulated and long processes where pathological and compensatory phenomenon may occur.Actually, data converge to demonstrate a complex dual role of RyR in AD acting as a potential compensatory/protective paradigm, or as a pathological hallmark amplifying the setting of histopathological lesions and synaptic deficits that are associated with the late AD stages. We provide evidences that RyR interfere with different routes leading to AD pathogenesis development through the amplification of APP metabolism and Aβ peptide production, the control of neuronal death, synaptic structure and plasticity dysfunctions and learning and memory decline (Figure 3).

About 30 millions individuals are estimated to be affected with AD worldwide and to date no effective treatment exists to arrest disease progression. Therapeutic approaches targeting Ca^2+^ influx have demonstrated efficacy in animal AD models but very few have been successful in clinical trials [174,175]. Targeting of ER Ca^2+^ homeostasis could be an additional therapeutic approach that merit testing. Data described above demonstrate that RyR could be envisaged as a potential new target. Therefore, we believe that it is of most interest to develop and test new RyR modulators with high specificity and affinity for RyR bioavailability as new therapeutic tools in AD.

## Abbreviations

Aβ: Amyloid β peptide; AD: Alzheimer Disease; AMPA: α-Amino-3-hydroxy-5-methyl-4-isoxazolepropionic acid; βAPP: β Amyloid precursor protein; Ca^2+^: Calcium; [Ca^2+^]_cyt_: Cytosolic calcium-concentration; [Ca^2+^]_ER_: Endoplasmic reticulum calcium-concentration; CICR: Ca^2+^-induced Ca^2+^ release; ER: Endoplasmic reticulum; IP_3_: Inositol 1,4,5-triphosphate; IP_3_R: Inositol 1,4,5-triphosphate receptor; NMDA: N-methyl-D-aspartate; PS: Presenilin; RYR: Ryanodine receptor; SERCA: Sarco-Endoplasmic reticulum Ca^2+^-ATPase; VGCC: Voltage gated Ca^2+^ channel.

## Competing interests

The authors declare that they have no competing interests.

## Authors’ contributions

DDP drafted the first version of this review. MC and FC revised the manuscript for intellectual content. All authors read and approved the final manuscript.
